# Continuous wound infiltration versus placebo following elective minimally invasive colorectal surgery (CIMICS): study protocol for a randomised controlled trial

**DOI:** 10.1371/journal.pone.0340859

**Published:** 2026-01-21

**Authors:** Sofie Glazemakers, Stijn H.J. Ketelaers, Harm J. Scholten, Robert-Jan Schipper, Michaël I. Meesters, Jacobus W.A. Burger, Johanne G. Bloemen

**Affiliations:** 1 Department of Surgery, Catharina Hospital, Eindhoven, the Netherlands; 2 GROW, Faculty of Health, Medicine, and Life Science, Maastricht University, Maastricht, the Netherlands; 3 Department of Anaesthesiology, Catharina Hospital, Eindhoven, the Netherlands; Medical University of Graz: Medizinische Universitat Graz, AUSTRIA

## Abstract

**Background:**

Enhanced Recovery After Surgery (ERAS) programs emphasize multimodal analgesia to minimize opioid use and improve patient outcomes. Continuous wound infiltration (CWI) with local analgesic is a promising adjunct to multimodal analgesia. However, its benefits in minimally invasive procedures and ERAS-adherent care remain unknown. This trial investigates whether the addition of CWI to standard ERAS care improves postoperative recovery following minimally invasive colorectal surgery.

**Methods:**

In this single-centre, blinded, randomised controlled trial, 192 eligible patients are randomised to receive either a CWI system with bupivacaine 0.125% (the interventional arm), or a placebo CWI with physiological saline (the control arm). All patients receive standardized ERAS perioperative care with multimodal analgesia. The primary outcome is the Quality of Recovery-15 score (QoR-15NL) on postoperative day 2. Secondary outcomes include QoR-15NL and pain scores (postoperative days 1–5), opioid consumption, length of hospital stay, key functional recovery milestones, and 90-day postoperative complications.

**Discussion:**

This will be the first randomised controlled trial evaluating the effect of CWI within minimally invasive and ERAS-adherent colorectal surgery. The trial findings may improve evidence-based perioperative care guidelines and enhance postoperative multimodal analgesia.

## Introduction

### Background and rationale

Enhanced Recovery After Surgery (ERAS) guidelines are evidence-based perioperative care pathways designed to optimise patient outcomes [1]. Through a multimodal approach, ERAS aims to minimise perioperative stress and accelerate post-operative recovery. In elective colorectal surgery, adherence to at least 70% of all ERAS elements is strongly associated with improved patient outcomes, fewer complications, shorter hospital stays, and decreased healthcare costs [2–4].

A key element of ERAS in colorectal surgery is multimodal pain management [1,5]. This strategy aims to maximise pain control through non-opioid alternatives to reduce opioid consumption and its associated side effects (e.g., sedative effects, nausea, ileus) [6–8]. A decrease in opioid use results in improved recovery, with earlier return of bowel function, fewer postoperative complications, and earlier postoperative discharge [7,9].

Despite the recommended opioid-sparing approach, opioids are often still required to achieve adequate pain control [1]. Novel strategies are therefore needed to further reduce reliance on opioids. Several locoregional analgesic techniques, including epidural blockades and abdominal wall blocks, have been established as effective, opioid-sparing additions to multimodal analgesia, especially in open colorectal surgery. However, these techniques are less suitable for minimally invasive surgery. For instance, epidural analgesia has been shown to potentially extend length of hospital stay in patients undergoing laparoscopic procedures [10,11]. In contrast, continuous wound infusion (CWI) of a local analgesic has not been linked to significant side effects that could impede recovery, therefore rendering it a promising local pain management strategy for minimally invasive colorectal surgery [12].

CWI has shown to be an effective and opioid-sparing adjunct in various abdominal surgeries, including open colorectal surgery [12–18]. However, studies evaluating CWI within minimally invasive and ERAS-adherent colorectal surgery are relatively scarce. In a prospective cohort study by the authors of the current protocol, the implementation of CWI in ERAS protocols was associated with minimal opioid use and enhanced recovery after surgery, resulting in excellent patient outcomes [19].

Given the promising effects and well-known safety profile, CWI is standard of care in some treatment centres, and recommended in the PROSPECT postoperative pain management guidelines [19,20]; however, its potential benefit remains to be proven in a randomised trial. This research therefore aims to evaluate CWI as an addition to the current multimodal analgesia.

### Objectives

The main objective of this single-centre randomised controlled trial is to investigate whether the addition of CWI with bupivacaine 0.125% (a local analgesic) to ERAS-adherent care improves patient recovery following minimally invasive colorectal surgery, compared to placebo (physiological saline). Additionally, the impact on postoperative pain scores, opioid consumption, length of hospital stay, and complications will be evaluated.

## Methods

### Study design and setting

This is a prospective single-centre, blinded, randomised controlled superiority trial with a 1:1 allocation ratio. This single-centre trial is being conducted at the Catharina Hospital, Eindhoven, the Netherlands.

**Fig 1:**
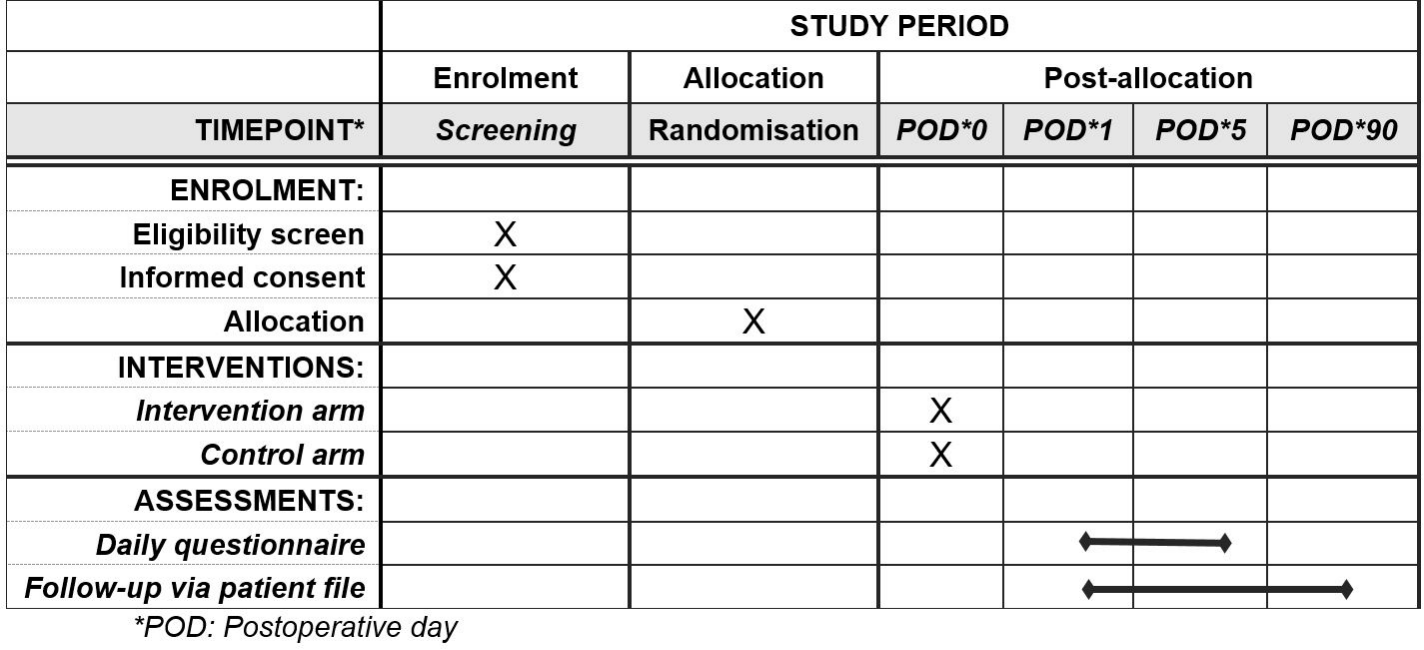
Standard protocol items: Recommendations for interventional trials (SPIRIT) enrolment schedule, interventions, and assessments.

**Fig 2:**
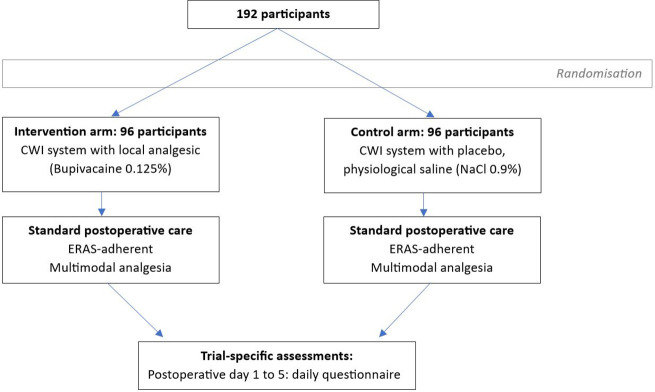
Study flowchart.

This trial is designed following the Standard Protocol Items: Recommendations for Interventional Trials (SPIRIT) guidelines. Figs 1 and 2 provide an overview of the study plan.

### Trial status

Ethical approval for this study was granted by the Medical Research Ethics Committees United – Nieuwegein (registration number W22.021) on July 11, 2024. The final protocol version is Version 3 (July 9, 2024), and the first patient was recruited on November 7, 2024. Recruitment is expected to be complete by November 2027. Data collection is expected to be complete by February 2028, and results are expected by April 2028.

### Eligibility criteria

Eligible patients are adults (age ≥ 18 years) scheduled for minimally invasive colorectal surgery with sufficient cognitive ability to provide written informed consent and to complete the questionnaire. The exclusion criteria are any contra-indication to the CWI-system or the local analgesic (e.g., allergy); inflammatory bowel disease (i.e. Crohn’s disease or ulcerative colitis); emergency surgery; or chronic opioid consumption (i.e., daily use for at least 90 days) or active drug addiction.

All patients scheduled for elective minimally invasive surgery in the study centre will be screened by their treating physician or physician assistant during their preoperative outpatient consultation. All eligible patients are informed about the study by their treating physician or physician assistant. Further information regarding the study will be provided by a research nurse or a local investigator if necessary. A written informed consent is required of all study participants before inclusion in the study. In cases where cognitive ability is in question, patients are referred to a multidisciplinary pathway, which includes cognitive screening by a geriatric specialist, as part of standard care.

### Interventions

This trial is designed to compare the addition of CWI with a local analgesic to the standard of care. Therefore, a placebo CWI system with physiological saline is used as comparator. Included patients will be randomised to receive either a CWI system containing 350 mL of bupivacaine 0.125% (intervention arm), or a CWI system containing 350 mL physiological saline, NaCl 0.9% (control arm).

#### CWI placement.

At the end of the surgical procedure, the CWI system is placed by a trained surgeon. The wound catheter is placed in the preperitoneal layer, a few centimetres from the Pfannenstiel incision. Following this placement and closure of the fascia and skin, all patients receive a 10 ml bolus of bupivacaine 0.125% through the wound catheter at the end of the procedure. The catheter is subsequently connected to the infiltration pump, which has been filled with 350 ml of either bupivacaine 0.125% or physiological saline, depending on the patients’ allocation. The standard infusion rate is set at 5 ml/h.

The continuous wound infusion is continued until the pump is empty. The system is removed by the patient when already discharged from the hospital. The CWI system may be removed prematurely if it is inadvertently displaced, or if it is the preference of the patient or treating physician, e.g., upon hospital discharge.

#### Standardized anaesthetic protocol.

All patients receive a standardized opioid-sparing anaesthetic regimen according to departmental practice. Induction is achieved with propofol (1.5–3 mg/kg), sufentanil (0.2–0.3 µg/kg), esketamine (0.25 mg/kg), lidocaine (1.5 mg/kg), and rocuronium (0.6 mg/kg). Anaesthesia is maintained using total intravenous anaesthesia with continuous propofol and based on the bispectral index (BIS), targeting BIS value between 40 and 60. Esketamine (0.1–0.2 mg/kg/h) and lidocaine (1.5 mg/kg/h) are continued as perfusions; sufentanil is titrated according to clinical requirement. Lidocaine infusions are discontinued approximately 45 minutes prior to placement of CWI to allow for a wash-out period.

#### Postoperative pain management.

All patients receive standard ERAS-adherent postoperative care according to standard practice. Postoperative pain is managed through a multimodal analgesic protocol. This includes a standard dose of paracetamol 1000 mg 3–4 times daily and a standard dose of metamizole 1000 mg 3 times daily during hospital stay. If pain control is inadequate, CWI infusion rate is adjusted from 5 ml/h to 8 ml/h. If pain control continues to be suboptimal, opioids can be administered according to the standard practice; starting with additional short-acting oxycodone as needed (as oxynorm 5 mg tablets with a maximum dose of 30 mg a day). If necessary, this is supplemented by long-acting oxycodone (as oxycontin 10 mg tablets with a maximum dose of 20 mg a day).

#### ERAS-adherent care.

All patients receive ERAS-adherent standard of care (see Table 1). ERAS compliance is measured and monitored by scoring adherence to each ERAS element to calculate total adherence.

**Table 1:** The enhanced recovery protocol after colorectal cancer surgery used in the study centre.

Preadmission patient education regarding the protocol	All patients receive extensive information and education with an informational and an educational consult with a specialised nurse.
Preadmission screening and optimisation for nutritional deficiency, frailty, tobacco cessation and alcohol use	Patients are screened for nutritional deficiency using the MUST or PG-SGA scoring system, and for frailty by using the G8 scoring system. Patients are referred preoperatively for tobacco and alcohol counselling. Anaemia screening is performed and, if applicable, intravenous iron replacement treatment is given.
Fasting and carbohydrate loading guidelines	Normal diet until 6h preoperatively, clear liquids until 2h preoperatively, preoperative carbohydrate treatment (400 ml Preop Nutricia) 2h preoperatively.
Pre-emptive medication (dose, route, timing) and antibiotics prophylaxis	1000 mg paracetamol oral in preoperative ward. No preoperative sedatives. Standard iv antibiotic prophylaxis within 15 min before incision.
Antiemetic prophylaxis (dose, route, timing)	1 mg granisetron and 4 mg dexamethasone given intravenously preoperatively.
Intraoperative fluid management strategy	Restrictive intraoperative fluid administration aimed at maintaining euvolemia. No advanced hemodynamic monitoring
Types, doses, and routes of anaesthetics administered	Continuous propofol, intravenous lidocaine, sufentanil, rocuronium, and low-dose ketamine infusion, no volatile anaesthesia. Anaesthesia was maintained based on the bispectral index (BIS), targeting between 40 and 60.
Patient warming strategy	Forced warm air blanket.
Management of postoperative fluids	Limited and guided by clinical requirement.
Postoperative analgesia and antiemetic plans	Standard dose of paracetamol 1000 mg 3–4 times daily, and a standard dose of metamizole 1000 mg 3 times daily during hospital stay. 3 times 1 mg granisetron or 3 times 10 mg metoclopramide on postoperative day 1, from day 2 only when necessary.
Plan for opioid minimization	No standard opioids. Short-acting opioids for breakthrough pain (fentanyl 100mcg on recovery; 5 mg oral oxycodone after return to ward).
Drain and line management	No routine wound drains, Foley catheter removed in OR. Infusion line stopped after retun to the ward and start of intake.
Early mobilisation strategy	Patients ambulate direct on surgical ward. At least getting out of bed on day 0, out of bed all meals, out of bed at least 6 h per day starting from postoperative day 1.
Postoperative diet and bowel regimen management	Clear liquids and regular diet beginning on postoperative day 0.
Criteria for discharge	Tolerating oral intake, independent diuresis, pain well controlled on oral medication, ambulating in hallways, no signs of complications.
Tracking of post-discharge outcomes	Follow-up by telephone on day 2 after discharge, or at-home telemonitoring. Follow-up at 1 week after discharge and at 1 month after discharge.

### Outcomes

Postoperative recovery will be measured and compared using the Dutch Quality of Recovery (QoR-15NL) questionnaire score. The Quality of Recovery score is a validated patient-reported measure that quantifies postoperative recovery through multiple factors such as pain levels, physical independence, nausea, and emotional state [21,22].

The primary outcome is the Dutch Quality of Recovery-15 (QoR-15NL) score on postoperative day 2. Postoperative day 2 specifically was selected *a priori* as the primary endpoint, as it reflects the early recovery period when differences in analgesic effectiveness are expected to be most pronounced and impactful.

Secondary outcomes include the following: (1) QoR-15NL scores on postoperative day 1–5; (2) Numeric Rating Scale (NRS) score of mean pain intensity on postoperative day 2 specifically, and from postoperative day 1–5; (3) total postoperative opioid use in milligrams of morphine equivalents; (4) postoperative length of stay in days; (5) parameters related to postoperative recovery, such as time to first stool passage, adequate pain control, mobilisation, and intake; and (6) postoperative complications within 90 days, graded according to Clavien-Dindo.

### Randomisation and blinding

Randomisation is managed by a local investigator using Castor EDC, with a computer-generated allocation sequence using block randomization with variable block sizes (4, 6, and 8) to allocate patients in a 1:1 ratio to the intervention or control arm.

Based on the allocated treatment arm, the CWI system is prepared with either local analgesic or placebo saline by a specialised (research) nurse or physician-scientist not involved in the patient’s care, labelled, and delivered to the preoperative holding. This procedure ensures that patients as well as treating physicians and nursing staff remain blinded to the allocation. Sufficient reason to unblind to patient allocation is left to the discretion of the treating physician and can be achieved by contacting the principal investigator.

## Data collection and management

### Assessment and collection of outcomes

Trial-specific assessments are limited to the conduction of a daily questionnaire from postoperative day 1–5. This questionnaire contains the Dutch Quality of Recovery-15 questionnaire, as well as 7 additional questions regarding postoperative analgesic use, pain scores, and functional recovery (see protocol addendum A). The questionnaire is either completed in writing on the treating ward and collected by the research team upon completion; or prompted and completed digitally via the electronic case report form (eCRF) of Castor EDC, with a standard timing in the late morning. Questionnaire completion is actively monitored by participating researchers, and discharged patients with open questionnaires are contacted by telephone in the late morning of the same day to ensure complete follow-up.

There are no further procedures or assessments for patients participating in this study that are additional to standard care. Additional information necessary for completion of the study will be collected from the patient file, where it is noted as part of standard medical care.

### Data management

Individual patient information obtained as a result of this study is considered confidential and is handled conform the Dutch Personal Data Protection Act (AVG) and the General Data Protection Regulation (EU) 2016/679. All data necessary for the analysis of the described endpoints is pseudonymized and collected in CRFs using Castor EDC, an ISO 27001-certified electronic data capture system. Source documents for this study will include hospital records and questionnaire forms completed in writing. Data which has not been pseudonymized can only be viewed by authorized personnel. On all study-specific documents other than the signed consent, the subject will be referred to by the study subject identification code.

## Statistical methods

### Statistical methods for primary and secondary outcomes

Statistical analysis will be performed using IBM SPSS Statistics (IBM Corporation and its licensors, 2017. Armonk, NY). A p-value of <0.05 will be considered statistically significant. Descriptive analyses will be used to describe baseline characteristics. Categorical data will be presented as frequencies and percentages, and continuous variables as means with standard deviations or medians with interquartile ranges as appropriate.

Primary outcome analysis will be done using unpaired t-tests or Mann-Whitney U tests, depending on the distribution of the data. Secondary outcome analyses will be performed depending on the distribution of data: (1) for continuous variables measured once: unpaired t-tests or Mann-Whitney U tests; (2) for continuous variables measured repeatedly: linear mixed model analyses or Friedman tests, with the p-value adjusted in line with the Bonferroni method; (3) for categorical variables: chi-squared tests or Fisher’s exact tests. All analyses will be based on the intention-to-treat principle. All participants will be included in the analysis as per their original group assignments.

No interim analysis will be conducted during this study period. No additional analysis, such as subgroup analysis, is intended for this study.

### Sample size

We hypothesize that the addition of CWI will lead to a clinically significant increase in the QoR-15NL score on postoperative day 2. Previous studies indicate that a clinically significant and relevant difference can be demonstrated with a difference in the QoR-15NL score of 6 points (with an SD of 14 points) [21].

Using this cut-off value for a sample size calculation with a Mann-Whitney U-test to account for possible non-normally distributed data, 91 patients are needed per randomization group to achieve 80% power with an alpha of 0.05 to detect a clinically-relevant difference in QoR-15NL score between CWI with local analgesics and placebo. Since a drop-out rate of 5% can be expected (e.g., conversion to open surgery), this study aims to include 96 patients per group, or 192 patients in total.

## Oversight and monitoring

### Adverse event reporting and harms

As this study is a low-intervention clinical trial, the reporting of adverse events uses a simplified risk proportionate approach. All adverse events Clavien-Dindo grade ≥2 within 48 hours of CWI-system removal, and all adverse events Clavien-Dindo grade ≥2 that may be linked to CWI-placement will be recorded in the CRF. All adverse effects will be reported in accordance with the applicable local and European Union laws and regulations.

### Data monitoring committee and auditing

Given that the safety profile of the investigational product is well-documented in literature and is already standard of care, no data monitoring committee has been formed. In accordance with the low-intervention classification of this trial, monitoring and auditing is conducted by an independent monitor from the study centre using a risk-based approach.

## Discussion

The CIMICS trial is the first randomised controlled trial evaluating CWI in ERAS-adherent minimally-invasive colorectal surgery with patient-reported recovery as primary endpoint.

As a non-opioid-based analgesic modality, CWI aligns closely with the multimodal analgesia principles recommended in ERAS guidelines [1]. Additionally, postoperative management and removal of CWI is technically straightforward and does not require specialised training or resource-intensive monitoring. Its safety profile has consistently been favourable: adverse effects are typically mild and localised (pain, redness, or swelling at the infusion site) [12,23]. The risk of local anaesthetic systemic toxicity when combining intravenous lidocaine and bupivacaine through CWI is minimised by using a standard low concentration of bupivacaine (0.125%) with a limited total administered dose, conservative lidocaine dosing, and the incorporation of a wash-out period between the discontinuation of intravenous lidocaine and the initiation of bupivacaine infusion [24]. Earlier theoretical concerns regarding increased wound complications or impaired healing with CWI usage have not been supported by empirical evidence; multiple studies and meta-analyses consistently report no elevated risk of infection or tissue recovery with CWI use [23,25]. Given this well-known safety profile and its promising effects, CWI use is already standard of care in select centres [19]. However, high-quality randomised evidence within a minimally invasive and ERAS-adherent setting is lacking.

While CWI has shown to be an effective adjunct in various open abdominal surgeries, its added value in the context of modern minimally invasive colorectal procedures and protocolised ERAS care remains uncertain [12–18,20]. Previous studies evaluating CWI’s analgesic benefit in minimally invasive colorectal surgery have produced inconsistent results. Fustran et al. and Oh et al. both reported a reduction in opioid consumption with the addition of CWI to intravenous patient-controlled analgesia, whereas the CATCH trial found no meaningful difference in pain scores or opioid consumption [18,26,27]. Additionally, although it has been hypothesised that implementation of CWI would have the most benefit within a strict ERAS protocol, no existing trials have evaluated CWI in ERAS-adherent care [19].

Additionally, no trials have evaluated CWI in colorectal surgery with patient-reported recovery as primary outcome. The rationale for the selection of the Quality of Recovery score as the primary outcome for this study was based on the multidimensional nature of early postoperative recovery. Ultimately, any benefits of CWI usage should translate to an improvement in patient recovery. Several validation studies have demonstrated the sensitivity and reliability of QoR-15NL in detecting clinically-relevant differences in postoperative recovery [28–30]. Along with secondary outcomes such as pain scores, opioid consumption, and functional recovery, QoR-15NL scores will enable a comprehensive assessment of the clinical impact of CWI.

The findings of this trial will inform clinical practice regardless of outcome. Positive findings would provide evidence-based support for integrating CWI into routine postoperative care. Conversely, no demonstrated benefit would argue against the addition of CWI to current multimodal analgesia, thereby avoiding an unnecessary intervention and simplifying postoperative care without compromising recovery. In either scenario, the results of the CIMICS trial will meaningfully inform perioperative care strategies in colorectal surgery, and contribute to their ongoing improvement.

### Potential limitations

Several potential limitations of this trial should be acknowledged. First, as this is a single-centre trial, external validity of findings may be limited. Secondly, there is potential for non-response bias, as the primary outcome is patient-reported; patients experiencing a poorer recovery may be less likely to complete the daily questionnaires. To minimize this, questionnaire completion is actively monitored, and paper questionnaires are provided on the ward for in-hospital patients. Furthermore, secondary outcomes include several measures of postoperative recovery, such as length of hospital stay, that are recorded regardless of questionnaire completion.

## Supporting information

S1 File Spirit checklist. See supporting file, separately submitted.
(DOC)

S2 File Protocol as approved by the ethics committee. See supporting file, separately submitted.
(PDF)

## Abbreviations

CIMICS: Continuous wound Infiltration versus placebo following elective Minimally Invasive Colorectal Surgery (CIMICS)
CRF: Case Report Form
CWI: Continuous Wound Infusion
eCRF: Electronic Case Report Rorm
ERAS: Enhanced Recovery After Surgery
NRS: Numeric Rating Scale
QoR-15NL: Dutch Quality of Recovery-15
