# Supplementary material for: Continuous wound infiltration versus placebo following elective minimally invasive colorectal surgery (CIMICS): study protocol for a randomised controlled trial
Source: PLoS One. 2026 Jan 21;21(1):e0340859. doi: 10.1371/journal.pone.0340859 (PMC12822994; doi:10.1371/journal.pone.0340859)
Supplement: S2 File — See supporting file, separately submitted. (PDF) [file pone.0340859.s002.PDF]

Continuous Wound Infiltration versus placebo following elective minimally invasive colorectal surgery; a double-blinded, randomised, placebo-controlled, low-intervention trial (CIMICS trial)

Continuous Wound Infiltration versus placebo following elective minimally invasive colorectal surgery; a double-blinded, randomised, placebo-controlled, low-intervention trial (CIMICS trial).

**Short title**	CIMICS trial
**EU trial number**	2024-512107-39
**Protocol version**	3
**Protocol date**	09-07-2024
**Sponsor**	Catharina Hospital<br>Michelangelolaan 2<br>5623 EJ Eindhoven
**Coordinating and Principal investigator**	Dr. J.G.Bloemen<br>Catharina Hospital<br>Department of Surgery<br>Michelangelolaan 2<br>5623 EJ Eindhoven<br>+3140 2396600<br>johanne.bloemen@catharinaziekenhuis.nl
**Participating investigators**	S.T. Glazemakers<br>Catharina Hospital<br>Department of Surgery<br>Michelangelolaan 2<br>5623EJ Eindhoven<br>sofie.glazemakers@catharinaziekenhuis.nl<br><br>Dr. H. Scholten<br>Catharina Hospital<br>Department of Anesthesiology and ICU<br>Michelangelolaan 2<br>5623 EJ Eindhoven<br>+3140 2398501<br>harm.scholten@catharinaziekenhuis.nlmailto:<br><br>Dr. S.H.J. Ketelaers<br>Catharina Hospital<br>Department of Surgery<br>Michelangelolaan 2<br>5623 EJ Eindhoven<br>stijn.ketelaers@catharinaziekenhuis.nl
**Funding party**	MSB Innovatiefonds<br>Coöperatie Medisch Specialistisch Bedrijf Catharina u.a.<br>Michelangelolaan 2<br>5623 EJ Eindhoven<br>+3140 2398518<br>msbhrm@catharinaziekenhuis.nl
**Independent expert (s)**	Dr. A.J.R. de Bie<br>Department of Intensive Care<br>Michelangelolaan 2<br>5623 EJ Eindhoven

PROTOCOL SIGNATURE SHEET

Name	Signature	Date
**Head of Department:**<br>A. van de Veen, MD, PhD<br>Department of Surgery<br>Catharina Hospital, Eindhoven<br><br>K. Bijleveld, MD<br>Department of Anaesthesiology<br>Catharina Hospital, Eindhoven		
**Coordinating and Principal Investigator:**<br>J.G. Bloemen, MD, PhD<br>Department of Surgery<br>Catharina Hospital, Eindhoven		

DOCUMENT HISTORY

Document	Date of version	Summary of Changes
**Original protocol, Version 1**	21-02-2024	Not applicable
**Version 2**	25-06-2024	Small adjustments based on RFI-CT-2024-512107-39-00-IN-003:<br>• Detailing the scoring of ERAS compliance;<br>• Including the use of drug accountability report forms;<br>• Adjusting the data retention period to 25 years;<br>• Detailing the protocol regarding use of the IMP label;<br>• Clarifying the anticipated drop-out rate;<br>• Incorporation of mixed-model and ANCOVA analysis in the secondary objectives;<br>• Accounting for multiplicity in case of repeated measures via the Bonferroni correction;<br>• Including the methods of handling missing data;<br>• Noting the measures that will be implemented in case of data security breach;<br>• Specifying at which timepoints the primary and secondary analysis endpoints will be compared; and at which timepoint the QoR scores are collected;<br>• Noting the expected mean QoR score in the interventional and control group;<br>• Removing mentions of the DSMB, superiority of CWI in the power calculcation, and legally designated representatives.
**Version 3**	09-07-2024	Chapter 16.6 (Data breach response procedures) has been added to this protocol, in response to RFI-CT-2024-512107-39-00-IN-005-01.

CONFIDENTIALITY STATEMENT

This document contains confidential information that must not be disclosed to anyone other than the sponsor, the investigative team, regulatory authorities, and members of the Research Ethics Committee.

TABLE OF CONTENTS

1. ABBREVIATIONS ... 7
2. SYNOPSIS ... 8
3. INTRODUCTION AND RATIONALE ... 10
3.1 Current treatment approach ... 10
3.2 Continuous Wound Infusion (CWI) ... 10
3.3 Protocol rationale ... 10
3.4 Justification of low-intervention classification ... 11
4. STRUCTURED RISK ANALYSIS ... 12
4.1 Potential issues of concern ... 12
4.2 Overall synthesis of the direct risks for the research subjects ... 12
5. OBJECTIVES AND ENDPOINTS ... 13
6. STUDY PLAN AND DESIGN ... 15
6.1 Trial Design ... 15
6.2 Sample size calculation and number of patients ... 15
6.3 Overall study duration and follow-up ... 16
7. STUDY POPULATION ... 17
7.1 Population ... 17
7.2 Inclusion criteria ... 17
7.3 Exclusion criteria ... 17
8. STUDY TREATMENTS ... 18
8.1 Investigational Medicinal Product (IMP) ... 18
8.2 Placebo ... 18
8.3 Additional considerations for trials involving a medical device ... 19
8.4 Preparation and labelling of the study treatment(s) ... 19
9. OTHER TREATMENTS AND RESTRICTIONS ... 20
10. TRACEABLILITY, STORAGE, ACCOUNTABLILITY AND COMPLIANCE ... 21
11. STUDY ASSESSMENTS AND PROCEDURES ... 22
11.1 Screening and recruitment ... 22
11.2 Randomisation, blinding and treatment allocation ... 22
11.3 Study procedures and assessments ... 22
12. STUDY DISCONTINUATION AND COMPLETION ... 24
12.1 Definition End of Trial ... 24
12.2 Criteria for temporary halt and early termination of the clinical trial ... 24
12.3 Discontinuation/withdrawal of individual subjects ... 24
13. SAFETY REPORTING ... 25
13.1 Definitions ... 25
13.2 Recording and reporting of AEs and SAEs ... 25
13.3 Follow-up of adverse events ... 26
13.4 Reporting of SUSARs by the sponsor to EudraVigilance ... 26

13.5Annual safety report 26
13.6Unblinding procedures for safety reporting 26
13.7Temporary halt for reasons of subject safety 27
13.8Urgent safety measures and other relevant safety reporting 27
13.9Data Safety Monitoring Board (DSMB)/Data Monitoring Committee (DMC)........ 27
14. STATISTICAL ANALYSIS........ 28
14.1Description of statistical methods 28
14.2Randomisation and blinding 28
14.3Sample size........ 28
14.4Primary analysis........ 28
14.5Secondary analysis 28
14.6Procedure for accounting for missing, unused and spurious data 30
15. ETHICAL CONSIDERATIONS 31
15.1Declaration of Helsinki 31
15.2Recruitment and informed consent procedures........ 31
15.3Benefits and risks assessment, group relatedness........ 31
15.4Compensation for injury 31
15.5Compensation for subjects 31
15.6Compensation for investigators........ 32
16. ADMINISTRATIVE ASPECTS, MONITORING AND CONFIDENTIALITY 33
16.1Approval initial application and substantial modifications........ 33
16.2Monitoring........ 33
16.3Recording, handling and storage of information........ 33
16.4Audits and inspections and direct access to source data/documents........ 34
16.5Reporting of serious breaches 34
16.6Data breach response procedures 34
16.7Notification of the start and the end of the recruitment 35
16.8Temporary halt/(early) termination........ 35
16.9Summary of the results 35
16.10Public disclosure and publication policy 35
17. REFERENCES 37
Addendum A........ 39

1. ABBREVIATIONS

ABR	General Assessment and Registration form (ABR form); in Dutch: Algemeen Beoordelings- en Registratieformulier (ABR-formulier)
AE	Adverse Event
AR	Adverse Reaction
CCMO	Central Committee on Research Involving Human Subjects; in Dutch: Centrale Commissie Mensgebonden Onderzoek
CRF	Case Report Form
CWI	Continuous Wound Infusion
DSMB	Data Safety Monitoring Board
eCRF	Electronic Case Report Form
ERAS	Enhanced Recovery After Surgery
EU	European Union
EudraCT	European drug regulatory affairs Clinical Trials
GCP	Good Clinical Practice
GDPR	General Data Protection Regulation; in Dutch: Algemene Verordening Gegevensbescherming (AVG)
GMP	Good Medical Practice
IB	Investigator's Brochure
IC	Informed Consent
IMP	Investigational Medicinal Product
IMPD	Investigational Medicinal Product Dossier
LOS	Length of hospital Stay
QoR	Quality of Recovery questionnaires; in Dutch: QoR-NL
(S)AE	(Serious) Adverse Event
SPC	Summary of Product Characteristics; in Dutch: officiële productinformatie IB1-tekst
SUSAR	Suspected Unexpected Serious Adverse Reaction
UAVG	Dutch Act on Implementation of the General Data Protection Regulation; in Dutch: Uitvoeringswet AVG

2. SYNOPSIS

Full title: Continuous Wound Infiltration versus placebo following elective minimally invasive colorectal surgery; a double-blinded, randomised, placebo-controlled, low-intervention trial (CIMICS trial).
EU trial number: 2024-512107-39

Rationale: Patient outcomes in an ERAS protocol for minimally invasive colorectal surgery can be improved by optimising multimodal analgesia to improve pain scores and minimise opioid consumption. In current clinical practice, opioid use is still often necessary to achieve adequate pain control, despite its side effects and associated decreased patient outcomes. This study aims to evaluate a promising pain management technique as addition to the current multimodal analgesia: Continuous Wound Infusion (CWI) with a local analgesic.

Objective: The main objective of this trial is to assess the efficacy of CWI in conjunction with standard multimodal pain management in enhancing patient recovery. Secondary objectives include evaluating the impact of CWI on postoperative pain scores, opioid consumption, length of hospital stay, complications, and parameters related to functional recovery.

Main trial endpoints: The primary endpoint is post-operative recovery, quantified through Quality of Recovery (QoR) scores as reported daily from the first until the fifth post-operative day.

Secondary trial endpoints: Secondary end points include post-operative pain levels measured via the Numeric Rating Scale (NRS), opioid usage, and markers of functional recovery from the first until the fifth postoperative day. Additional secondary endpoints noted at 90-day follow-up are total length of hospital stay, condition at discharge and post-operative complications or re-admissions.

Trial design: This is a monocentre, double-blind, randomised, placebo-controlled, low-intervention trial. Eligible patients are randomised to receive either a CWI system with bupivacaine (the interventional arm), or a placebo CWI with physiological saline (the control arm) at the end of the surgical procedure.

Trial population: Adult patients scheduled for elective minimally invasive colorectal surgery at the research centre, excluding patients with inflammatory bowel disease or contra-indications to the intervention.

Interventions: In the interventional arm, patients receive a CWI system containing 350 mL of 1.25mg/mL bupivacaine at the end of the surgical procedure. In the control arm, patients receive a CWI system containing 350 mL physiological saline (NaCl 0.9%). The standard infusion rate for both groups will be set at 5mL/h, which will continue until the CWI system is emptied or removed. Trial-related monitoring procedures are limited to brief daily questionnaires.

Ethical considerations relating to the clinical trial including the expected benefit to the individual subject or group of patients represented by the trial subjects as well as the nature and extent of burden and risks:

The trial anticipates benefits for subjects in the interventional group through potential reduction in postoperative opioid use, enhanced pain control, and faster recovery. Excluding this, no altered post-operative course is expected for the interventional or control group.

Trial-specific burden is minimal, as it is limited to completing a brief daily questionnaire. Trial-specific risk is low, as the investigational medicinal product is standard of care. It has a well-documented and established safety profile and will be used in the authorised form for its authorised indication. The risk of technique-related complications is exceptionally low and evenly distributed across both groups.

3. INTRODUCTION AND RATIONALE

3.1 Current treatment approach

Enhanced Recovery After Surgery (ERAS) guidelines are evidence-based perioperative care pathways designed to optimise patient outcomes.[1] The ERAS protocol aims to minimise perioperative stress and accelerate post-operative recovery through a multimodal approach. In elective colorectal surgery, adherence to at least 70% of all ERAS elements is strongly associated with improved patient outcomes, fewer complications, shortened length of stay, and decreased healthcare costs.[2-4]

A key element within the ERAS protocol for elective colorectal surgery is adequate multimodal pain management.[1,5] Multimodal analgesia aims to maximise pain control achieved with non-opioid alternatives, in order to reduce opioid consumption and its associated side effects (e.g. sedative effects, nausea, ileus).[6,7,8] Previous studies have shown that diminished opioid use results in enhanced recovery, with earlier return of bowel function, fewer postoperative complications, and earlier discharge from the hospital.[7,9]

Despite the opioid-sparing approach, opioid use is still often necessary to achieve adequate pain control.[1] Novel strategies are needed to further improve non-opioid based multimodal analgesia and mitigate the need for opioids. Several methods are already used as additions to multimodal analgesia for this purpose, but scientific substantiation is often still lacking. This research aims to evaluate a promising pain management technique as an addition to the current multimodal analgesia: post-operative continuous wound infusion (CWI) with a local analgesic.

3.2 Continuous Wound Infusion (CWI)

Post-operative CWI with a local analgesic may function as an addition to multimodal pain management by alleviating local wound pain. In previous studies, CWI is considered an effective additional method of pain relief in a multitude of abdominal surgeries and proved at least as effective as epidural analgesia.[10-14] In addition, CWI has been shown to reduce opioid consumption following open colorectal surgery.[15-17]

Local and regional analgesia has previously been proven to be well-suited additions to ERAS-adherent multimodal analgesia. Epidural blockades and abdominal wall blocks are well-documented as opioid-sparing additions in enhanced recovery after open colorectal surgery. Unfortunately, such techniques are less suitable for pain management following minimally invasive surgery. Epidural analgesia, for example, has been shown to potentially extend length of hospital stay in those who underwent laparoscopic surgery.[12] CWI, on the other hand, has not been associated with any significant side effects that may hamper recovery. CWI is therefore considered a promising local pain management technique following minimally invasive colorectal surgery.[13]

Studies regarding the effectiveness of CWI within an ERAS protocol are relatively scarce. In a prospective cohort study by the authors of the current protocol, the implementation of CWI in ERAS protocols was associated with minimal opioid use and enhanced recovery after surgery, resulting in excellent patient outcomes.[18] These promising outcomes remain to be proven in a randomised trial. In the centre, the use of CWI following minimally invasive colorectal surgery is standard practice and has had good results.[18]

3.3 Protocol rationale

In conclusion, the addition of CWI to a multimodal analgesic protocol following minimally invasive colorectal surgery seems a promising method to enhance postoperative recovery. Although previous studies have demonstrated a beneficial effect of this modality on pain management and patient

outcomes, conclusive evidence remains lacking, in particular within an ERAS pathway. Given the promising effects and well-known safety profile, utilization of CWI in multimodal analgesia is standard of care in some treatment centres, including the study centre. Further studies are needed to determine the role of CWI in multimodal analgesia to improve postoperative recovery after minimally invasive colorectal surgery in an ERAS environment.

This study randomises patients who undergo minimally invasive colorectal surgery to receive either CWI with local analgesics (the interventional arm), or placebo CWI, filled with physiological saline (NaCl 0.9%) (the control arm). Patient outcomes will be recorded through a daily questionnaire containing the Quality of Recovery (QoR) questionnaire and 7 additional questions regarding functional recovery (see addendum A). The QoR has been developed as a standard measure to assess the early postoperative health status. It integrates recovery factors such as pain levels, capacity to ambulation, and nausea to provide a quantification of recovery, with a higher QoR score corresponding to a better recovery.[19] The 15-item scale version of the QoR (the QoR-15) ranges from a minimum score of 0 to a maximum score 150. It has been validated in multiple languages, including Dutch (QoR-15NL).[20]

The investigators hypothesise that the addition of CWI with a local anaesthetic to multimodal analgesia (the interventional arm) will result in an increase of the QoR score compared to treatment with the current multimodal pain management (the control arm). In addition, the investigators hypothesise reduced opioid use and shorter lengths of hospital stay in the interventional arm when compared to the control arm.

3.4 Justification of low-intervention classification

Given that (1) all investigational medicinal products are authorized, as well as (2) used in accordance with this authorization, and (3) the additional diagnostic procedures pose minimal additional burden and no additional risk to the safety of the participating patients as compared to normal clinical practice, this clinical trial may be classified as a low-intervention clinical trial (see Chapter 4).[25]

4. STRUCTURED RISK ANALYSIS

4.1 Potential issues of concern

Not applicable, as the investigational medical product will be used in the authorised form for the authorised indication; see Chapter 4.2.

4.2 Overall synthesis of the direct risks for the research subjects

The additional risk in all treatment arms of this study is limited to possible side effects related to the use of the CWI system. These include mild pain, redness, and swelling at the site of infusion, as well as a very low risk of infection at the site of infusion.[23] The risk of any complications occurring as result of this technique is very low; most articles do not report any complications at all (see Chapter 8.1). All mentioned potential risks as well as potential postoperative complications will be monitored during the patients' hospital stay, as well as during their follow-up appointments.

The risk of developing complications is as low in the intervention group as it is in the control group. The use of 'sham blocks' with saline is very common in literature. The use of this CWI system is necessary in both the intervention and the control group. It is the only effective way to reduce bias. Not performing a saline block in the control group would severely reduce the validity of this research.

Interventional treatment may reduce patient postoperative opioid consumption and accelerate recovery, which would be beneficial for the patients' comfort and overall outcomes of the ERAS protocol. The participating investigators consider the extra potential risk for patients in the interventional arm to be minimal and expect the risk of side effects to be outweighed against the potential benefit.

Given that (1) all investigational medicinal products are authorized, as well as (2) used in accordance with this authorization, and (3) the additional diagnostic procedures pose minimal additional burden and no additional risk to the safety of the participating patients as compared to normal clinical practice, this clinical trial may be classified as a low-intervention clinical trial (see Chapter 6.1.2).

5. OBJECTIVES AND ENDPOINTS

**Objectives**	**Endpoint(s)**
**Primary objective(s)**	**Endpoint for the primary objective(s)**
To determine the effect of continuous wound infusion with local analgesics on immediate post-operative recovery on postoperative day 2 for patients who underwent minimally invasive colorectal surgery.	Postoperative recovery will be measured and recorded using the Quality of Recovery (QoR) Questionnaire score, conducted daily from the first postoperative day until the fifth postoperative day (see addendum A). The questionnaire will be prompted and completed digitally using the electronic case report form (eCRF) of an ISO 27001 certified study database. For the analysis of the primary objective, only scores from the second postoperative day will be utilized.
**Secondary objective(s)**	**Endpoint(s) for the secondary objective(s)**
To compare post-operative trajectory from day 1 to day 5 between the interventional and control group.	Postoperative recovery will be measured and recorded using the Quality of Recovery (QoR) Questionnaire score, conducted daily from the first postoperative day until the fifth postoperative day (see addendum A). The questionnaire will be prompted and completed digitally using the electronic case report form (eCRF) of an ISO 27001 certified study database.
To compare postoperative pain levels between the interventional and control arm on postoperative day 2 specifically; and to compare the postoperative trajectory of pain levels from day 1 to day 5.	Postoperative pain levels will be measured and recorded using the Numeric Rating Scale (NRS), contained within the daily questionnaire (see addendum A). The questionnaire will be prompted and completed digitally using the electronic case report form (eCRF) of an ISO 27001 certified study database.<br>Additionally, NRS score will be registered daily by nursing staff during postoperative hospital stay, as is standard in the study centre.
To compare total postoperative opioid use between the interventional and control arm.	Postoperative analgesic use will be recorded within the daily questionnaire (see addendum A). The questionnaire will be prompted and completed digitally using the electronic case report form (eCRF) of an ISO 27001 certified study database.<br>Additionally, administration of any medication (including administration of opioids) will be registered by the treating physician, as is standard in the study centre. Opioid consumption will be converted to milligrams (mg) of morphine equivalents.
To compare postoperative length of stay and time until functional recovery between the interventional and control arm.	Date of discharge and functional recovery will be noted in the patient file by the treating physician. Functional recovery is defined as:<br>o Adequate pain control with oral analgesics only (except CWI)<br>o Independency in activities of daily living

	o Oral intake of at least 50% of normal caloric intake<br>o No intravenous fluid administration<br>o Temperature <38,5 degrees Celsius<br>o No signs of infectious complications<br>o Independent ostomy self-care by patient or involvement of home care services<br>o At least passage of flatulence
To compare incidence of postoperative complications between the interventional and control arm.	Possible complications will be included from date of surgery up to 90 days after surgery and are registered in the patient file by the treating physician. Complications will be graded according to Clavien-Dindo. These data will be extracted from de EIAS database as well as the patient file.
To compare parameters related to postoperative recovery, such as time until first flatulence and stool passage, mobilisation, intake, intravenous fluid administration, and condition at discharge between the interventional and control arm.	These markers of recovery will be noted by the nursing staff in the patient file, as is standard in the study centre, and will additionally be included in the daily questionnaire (see addendum A).
To compare compliance to the ERAS protocol between the interventional and control arm.	ERAS compliance data will be measured by scoring adherence to each ERAS element with a yes or no. The percentage of ERAS elements followed in the patient's peri-operative care is then calculated to determine overall compliance, as well as pre-, intra-, and post-operative adherence.
To assess the impact of the duration of CWI infusion on recovery outcomes.	Postoperative recovery will be measured and recorded using the Quality of Recovery (QoR) Questionnaire score, incorporated in the daily questionnaire conducted from the first postoperative day until the fifth postoperative day (see addendum A).<br>Duration of CWI infusion will be measured and recorded using the additional questions in the daily questionnaire.<br>The questionnaire will be prompted and completed digitally using the electronic case report form (eCRF) of an ISO 27001 certified study database.

6. STUDY PLAN AND DESIGN

6.1 Trial Design

This is a monocentre, double-blinded, randomised, placebo-controlled, low-intervention trial. Patients who are scheduled for minimally invasive colorectal surgery will be randomised in a 1:1 ratio to receive CWI with local analgesics (the interventional arm) or placebo CWI with physiological saline (the control arm).

6.1.1 Justification of placebo usage

As mentioned above, postoperative pain management after colorectal surgery is crucial for optimising outcomes. In an earlier prospective observational study, we found evidence that a CWI with local analgesics is related to low pain scores and minimal morphine consumption.[16] This may ultimately lead to less morphine induced side-effects and improved patient satisfaction. However, the earlier study was a prospective observational cohort study without a control group, and therefore did not compare outcomes with a control group and included a risk of bias. To determine the effect of CWI with local analgesics in colorectal surgery, a randomised controlled trial would offer more valuable scientific evidence.

The risk of complications of this technique is very low. Most articles do not report any complications. The risk of introducing complications is as low in the intervention group as in the control group. The use of 'sham blocks' with saline is very common in literature. This is the only effective way to reduce bias. Not performing a saline block in the control group would severely reduce the validity of this research.

6.1.2 Justification of low-intervention classification

The investigational product has a well-documented and well-established safety-profile, the use of which is routine clinical practice.[23] This study fulfils all necessary conditions to be classified as a low-intervention clinical trial set out by the EU Clinical Trial Regulation 536/2014 (CTR):[25]

- All investigational medicinal products are authorized (see Chapter 6);
- All investigational medicinal products will be used in authorised form for the authorised indication, and the use of the investigational medicinal product is supported by previous research demonstrating safety and efficacy (see Chapter 3 and 8).
- The additional diagnostic procedure, the completion of brief daily questionnaires (see addendum A), pose minimal additional burden and risk to the safety of the participating patients as compared to normal clinical practice (see Chapter 4).

6.2 Sample size calculation and number of patients

The primary endpoint of this study is the postoperative QoR score. We hypothesize that the addition of CWI will lead to a clinically significant increase in the QoR score. Based on previous studies, a clinically significant and relevant difference can be demonstrated with a difference in the QoR score of 6 points (with an SD of 14 points).[21,22]

Using this cut-off value for a sample size calculation with a Mann-Whitney U-test to account for possible non-normally distributed data, we need 91 patients per randomization group to achieve 80% power with an alpha of 0.05 in order to detect a clinically relevant difference in QoR score between CWI with local analgesics and placebo. We anticipate a drop-out rate of 5%. This accounts for potential cases in which the minimally invasive procedure is converted to open surgery. Such a conversion would disqualify the previously-included patient based on our inclusion and exclusion criteria. The estimated drop-out rate of 5% is based on the previous experiences at our research centre and slightly overestimated to accommodate any unforeseen variations from past outcomes.

Since we expect a drop-out rate of 5%, we aim to include 96 patients per group, or 192 patients in total.

6.3 Overall study duration and follow-up

Participating patients will receive either CWI with bupivacaine (the interventional arm), or placebo CWI with physiological saline (the control arm) upon completion of surgery, which may remain situated until the CWI system is empty or removed on indication (typically approximately 2 days). Follow-up will be concluded at 90 days post-operatively (see Chapter 5).

7. STUDY POPULATION

7.1 Population

This study will include all eligible patients undergoing elective minimal invasive colorectal surgery in the study centre. Over the past year, approximately 175 patients in the Catharina Hospital underwent minimally invasive colorectal surgery. Of these patients, it is expected that approximately 5% will not be eligible due to exclusion criteria. This leaves approximately 166 patients potentially eligible for inclusion each year. Assuming that 80-90% will be able to be included in the study, this will lead to an accrual rate of 133 patients per year.

7.2 Inclusion criteria

In order to be eligible to participate in this study, a subject must meet all of the following criteria:

- 18 years or older
- Scheduled for minimally invasive colorectal surgery
- Provided written informed consent

7.3 Exclusion criteria

A potential subject who meets any of the following criteria will be excluded from participation in this study:

- Any contra-indication to the CWI system or allergies for the used local analgesic
- Inflammatory Bowel Disease: ulcerative colitis or Crohn’s disease
- Emergency surgery
- Chronic opioid consumption (i.e. daily use for at least 90 days), or active drug addiction

8. STUDY TREATMENTS

8.1 Investigational Medicinal Product (IMP)

8.1.1 Name and description of the IMP

The investigational product used in this study is bupivacaine. The investigational medicinal product is prepared for use as IMP within the participating hospital. The following medicinal product is used in this preparation:

- Generic name: bupivacaine hydrochloride 0.25% w/v solution for injection.
- Trade name: Bupivacaine Eugia 2,5 mg/ml (RVG 20949, chemical name N01BB01).
- Formulation: solution for injection

The preparation of the investigational medicinal product includes the dilution of bupivacaine hydrochloride from a concentration of 0.25% to a concentration of 0.125%, as well as the insertion of the IMP within the CWI-system and the subsequent labelling of this system (see Chapter 8.4). The use of bupivacaine in a concentration of 0.125% is standard practice in the participating hospital.

The IMP will be supplied from commercial stock, in its authorized form and for its authorized indication. Prior to the preparation and labelling of the CWI, the drug accountability record form will be updated with the following information: drug name, batch number, patient name, patient study number, date of issue, date of expiry, initials for issue. No additional documentation regarding the manufacturing and import of the investigational medicinal product is necessary, given that the preparation of the investigational medicinal product takes place within a hospital legally authorised to carry out this process, and given that the investigational medicinal product is intended to be used exclusively in this same hospital.

8.1.2 Status of development of the IMP

Given the extensive experience with bupivacaine in human beings, clinical studies are not discussed in this protocol. The Summary of Product Characteristics can be found here[23].

8.1.3 Description and justification of dosage and route of administration

The investigational medicinal product is used in its authorised form, for its authorised indication. Bupivacaine will serve as a local anaesthetic, and will be administered through the CWI-system. Upon completion of the surgery and placement of the wound catheter, patients will receive a one-time administration of 10 ml bupivacaine 1.25 mg/mL through the catheter. The wound catheter will subsequently be connected to the infiltration pump, which has been filled with 350ml bupivacaine 1.25mg/mL by the anaesthetic team. The standard infusion rate will be set at 5 ml/h (150mg/24h) but can be adjusted to 3 ml/h (90mg/24h) or 8 ml/h (240mg/24h) depending on the patients' pain scores. Infusion will be continued until the pump is empty or the CWI system is removed.

When used in this manner, the dosage of the investigational medicinal product is well within the authorised maximum dosage for continuous infusion (400mg/24h).[23]

8.2 Placebo

This study includes a control arm. Patients randomised to the control arm receive a CWI-system filled with physiological saline (NaCl 0.9%; RVG 027512) (see Chapter 11.4.1). The use of placebo is necessary to determine the efficacy of the investigational intervention (see Chapter 6.1.1). The use of 'sham blocks' with saline is very common in literature. Patients receiving placebo CWI are not subject to any risk of serious or irreversible harm, or any additional risk when compared to the control group (see Chapter 6.1.1).

All patients, including patients randomised to the control arm, will receive standard post-operative care, which includes multiple other revenues of pain management in addition to the CWI-system (see Chapter 11.3.2). Patients in the control arm thus receive standard of care, as do patients in the investigational arm.

8.3 Additional considerations for trials involving a medical device

This study includes the use of a Continuous Wound Infusion (CWI) system. This CWI-system encompasses the following:

- The wound catheter; specifically, Infiltralong, as produced by Pajunk
- The infiltration pump; specifically, Fuserpump, as produced by Pajunk

The CWI-system is CE-marked and will be used in accordance with its intended use.

8.4 Preparation and labelling of the study treatment(s)

As described in Chapter 8.1.1, the investigational medical product is prepared for use within the participating hospital. This process includes the dilution of bupivacaine hydrochloride from a concentration of 0.25% to a concentration of 0.125%, as well as the insertion of the IMP within the CWI-system and the subsequent labelling of this system. Labelling of the study treatment is therefore limited to in-hospital relabelling of hospital-acquired and in-hospital-prepared medication. This will be done by a specialised (research) nurse or a physician-scientist, authorized for and familiar with this action, and not involved in the patient's care.

The CWI-system will be clearly labelled as containing CIMICS-trial medication with the patient's name, date of birth, and patient number, and delivered to the preoperative holding. This procedure ensures that patients as well as treating physicians and nursing staff are blinded for the randomisation. The CWI system will be placed by a trained surgeon at the end of the surgical procedure (see Chapter 11.4.1).

The IMP will be supplied from commercial stock, in its authorized form and for its authorized indication. Prior to the preparation and labelling of the CWI, the drug accountability record form will be updated with the following information: drug name, batch number, patient name, patient study number, date of issue, date of expiry, initials for issue. No additional documentation regarding manufacturing or import authorization is necessary, given that this methodical preparation of the investigational product is in compliance with GMP and GDP principles, and given that it concerns in-hospital relabelling of EU-authorized hospital-acquired medication that will consequently be used in-hospital.

9. OTHER TREATMENTS AND RESTRICTIONS

There are no additional specifically restricted or specifically permitted medications or lifestyle factors before or during the study.

10. TRACEABLILITY, STORAGE, ACCOUNTABLILITY AND COMPLIANCE

The IMP will be supplied from commercial stock, in its authorized form and for its authorized indication. Prior to the preparation and labelling of the CWI, the drug accountability record form will be updated with the following information: drug name, batch number, patient name, patient study number, date of issue, date of expiry, initials for issue. The drug accountability record form Additional documentation is not necessary, given that:

- This is a low-interventional clinical trial;
- The investigational medicinal product is authorised for the intended use;
- The investigational medicinal product is acquired from hospital stock
- Standard, normal prescribing practice and documentation applies;
- Specific documentation of the prescribed amounts and doses taken is available in the patient's medical records, as:
    - All patients receive a CWI-system, the contents of which are standardised according to the patient's allocated treatment arm (see Chapter 11.3.1);
    - Adjustments to the standard infusion rate and removal of the CWI-system are routinely noted in the patient's medical record;

11. STUDY ASSESSMENTS AND PROCEDURES

11.1 Screening and recruitment

All eligible patients scheduled for elective minimally invasive surgery in the study centre will be informed about the study by their treating physician or physician assistant. If a patient meets the in- and exclusion criteria, the treating physician will ask the patient to participate in the study. A participating investigator or research nurse will further inform the patient of the study in more detail, and provide the patient information form. The patient information form contains the contact details of the study team in case of additional questions from the patient.

After a sufficient amount of time, patients will be asked to participate in the study. Written informed consent will be signed in two-fold and dated by hand at the outpatient department by the patient and either the treating physician, the principal investigator, or physician assistant. After informed consent, the contact information of the patient will be sent to the coordinating investigator with the purpose of registering participation, allocating patients to a treatment arm, and providing post-operative questionnaires. If patients decide not to participate, they will receive standard of care and further treatment will not be affected by their decision.

11.2 Randomisation, blinding and treatment allocation

Eligible patients who signed informed consent will be randomised by the participating investigators. Randomisation will be performed by a central automatic randomisation tool with an intervention:control ratio of 1:1. Each randomised patient is assigned a sequential subject number.

The investigational product used in this study is a CWI system prepared with bupivacaine or physiological saline based on the allocated treatment arm. Preparation of the investigational product involves the in-hospital relabelling of hospital-acquired medication. This will be done by a specialised (research) nurse or a physician-scientist, authorized for and familiar with this action, and not involved in the patient’s care.

The CWI-system will be clearly labelled as containing CIMICS-trial medication with the patient’s name, date of birth, and patient number, and delivered to the preoperative holding. This procedure ensures that patients as well as treating physicians and nursing staff are blinded for the randomisation. The CWI system will be placed by a trained surgeon at the end of the surgical procedure (see Chapter 11.4.1).

Sufficient reason to unblind to patient allocation is left to the discretion of the treating physician. The logged medication can be found within the digital patient database if the treating physician explicitly indicates a necessity for this. The treating physician will inform the participating investigators of any unblinding.

11.3 Study procedures and assessments

11.3.1 CWI placement

At the end of the surgical procedure, a CWI system will be placed by a trained surgeon. The wound catheter will be installed in the preperitoneal layer, a few centimetres from the Pfannenstiel incision. Following this placement, all patients will receive a one-time administration of 10 ml bupivacaine 1.25mg/mL through the wound catheter. The catheter will subsequently be connected to the infiltration pump, which has been filled with 350ml of either bupivacaine or saline, depending on the patients’ allocation. The standard infusion rate will be set at 5 ml/h but can be adjusted to 8 ml/h depending on the patients’ pain scores. The continuous wound infusion will be continued until the pump is removed or empty (upon which standard care includes removing it as well).

11.3.2 Post-operative pain management

Post-operative care is the same for all patients, and according to the standard of care. A multimodal analgesic strategy will be employed. This includes a standard dose of paracetamol 1000mg 3-4 times daily and a standard dose of metamizole 1000 mg 3 times daily during the first three postoperative days or as long the patient is in the hospital. If pain control is inadequate, the infusion rate of the CWI pump will be adjusted from 5 ml/h to 8 ml/h. If pain control continues to be suboptimal, opioids can be administered according to the standard practice; starting with additional short-acting oxycodone as needed (as oxynorm 5mg tablets with a maximum dose of 30mg a day). In case of inadequate pain control despite this, long-acting oxycodone will be added (as oxycontin 10mg tablets with a maximum dose of 20mg a day).

11.3.3 Efficacy assessments

Trial-specific assessments are limited to daily questionnaires, which will be prompted and completed digitally from the first until the fifth postoperative day (see Chapter 5). This questionnaire contains the QoR-15, as well as 7 additional questions regarding functional recovery (see addendum A). Previous research has recorded a mean QoR score of 115 following colorectal surgery; we therefore anticipate a QoR score of 115 in the control arm, and a QoR score of 121 in the interventional arm.[26] The questionnaire will be prompted at 11 am every day through the electronic case report form (eCRF) of an ISO 27001 certified study database. It can then be completed digitally. For the analysis of the primary objective, only scores from the second postoperative day will be utilized.

There are no further procedures or assessments for patients participating in this study that are additional to standard care. Additional information necessary for completion of the study will be collected from the patient file, where it is noted as part of standard medical care (see Chapter 5).

11.3.4 Safety assessments

Not applicable, since all treatment options in this low-intervention clinical trial are considered standard of care. The interventional product will be used in the authorised form for the authorised indication. The focus of this research will be analysing the effectiveness as additions to current clinical practice. The safety profile of this intervention is already well-documented. Additional risk to participating patients is considered minimal (see Chapter 4 and 8).

12. STUDY DISCONTINUATION AND COMPLETION

12.1 Definition End of Trial

The end of this clinical trial is defined as 90 days after the primary operation of the last patient.

In accordance with Article 37, Chapter VI, of the CTR, the sponsor shall notify each Member State concerned of the end of a clinical trial in relation to that Member State through the EU portal. That notification shall be made within 15 days from the end of the clinical trial in the last of the Member States concerned and third countries in which the clinical trial has been conducted.

Irrespective of the outcome of a clinical trial, within one year from the end of a clinical trial in all Member States concerned, the sponsor shall submit to the EU database a summary of the results of the clinical trial, the content of which is set out in CTR Annex IV. It shall be accompanied by a summary written in a manner that is understandable to laypersons.

12.1.1 Justification of End of Trial

The end of this clinical trial is defined as 90 days after the primary operation of the last patient, rather than the date of the last visit of the last patient, due to the defined objectives and endpoints, which includes incidence of postoperative complications from date of surgery up to 90 days after surgery.

12.2 Criteria for temporary halt and early termination of the clinical trial

In accordance to section 10, subsection 4, of the WMO, the sponsor will suspend the study if there is sufficient ground that continuation of the study will jeopardise subject health or safety. The temporary halt or early termination of a clinical trial for reasons of a change of the benefit-risk balance shall be notified to the Member States concerned through the EU portal. That notification shall be made without undue delay but not later than in 15 days of the date of the temporary halt or early termination. It shall include the reasons for such action and specify follow-up measures. The study will be suspended pending a further positive decision by the concerned member state. The investigator will take care that all subjects are kept informed. The restart of the clinical trial following a temporary halt for reasons of a change of the benefit-risk balance shall be deemed to be a substantial modification subject to the authorisation procedure laid down in CTR Chapter III.

12.3 Discontinuation/withdrawal of individual subjects

Subjects can leave the study at any time for any reason if they wish to do so without any consequences. The withdrawal of the informed consent shall not affect the activities already carried out and the use of data obtained based on informed consent before its withdrawal. The investigator can decide to withdraw a subject from the study for urgent medical reasons. Individual patients will not be replaced after withdrawal. Potential withdrawal of patients is included in the drop-out rate of 5%. Randomised patients withdrawn from the treatment are included in the intention-to-treat population and analyses.

13. SAFETY REPORTING

13.1 Definitions

13.1.1 Adverse events (AEs)

Adverse events are defined as any untoward medical occurrence in a subject to whom a medicinal product is administered and which does not necessarily have a causal relationship with this treatment.

13.1.2 Serious adverse events (SAEs)

Serious adverse event is any untoward medical occurrence in a patient or trial subject that at any dose:

- results in death,
- is life-threatening,
- requires inpatient hospitalization or prolongation of existing hospitalization,
- results in persistent or significant disability/incapacity,
- is a congenital anomaly/birth defect

An elective hospital (re)admission will not be considered as a serious adverse event.

13.1.3 Suspected unexpected serious adverse reactions (SUSARs)

Unexpected adverse reactions are SUSARs if the following three conditions are met:

1. The event must be serious;
2. There must be a certain degree of probability that the event is a harmful and an undesirable reaction to the medicinal product under investigation, regardless of the administered dose;
3. The adverse reaction must be unexpected, that is to say, the nature and severity of the adverse reaction are not in agreement with the product information as recorded in the reference safety information (RSI).

13.2 Recording and reporting of AEs and SAEs

The recording and reporting of AEs and SAEs is simplified, using a risk proportionate approach, given that:

- This is a low-intervention clinical trial (see Chapter 6.1.2);
- The investigational medical product is authorized for the intended use (see Chapter 4 and 8);
- The IMP has a well-established safety profile.[23]

The investigator will record and report the following AE's and SAE's reported spontaneously by the subject or observed by the investigator or his staff:

- All AE's and SAE's Clavien-Dindo grade ≥2 occurring within 48 hours after the CWI-system is removed;
- All AE's and SAE's Clavien-Dindo grade ≥2 occurring within the follow-up period of 90 days that may be linked to placement of the wound catheter (e.g. infection at the site of infusion).

AE's and SAE's that occur after the CWI-system has been removed for 48 hours and that cannot be linked to placement of the wound catheter are not recorded, as the IMP has a limited half-life and a well-established duration of action that falls well within this 48-hour period.

The investigator shall report all relevant serious adverse events to the sponsor without undue delay but not later than within 24 hours of obtaining knowledge of the events. Where relevant, the investigator shall send a follow-up report to the sponsor to allow the sponsor to assess whether the serious adverse event has an impact on the benefit-risk balance of the clinical trial.

The sponsor shall keep detailed records of all adverse events reported to it by the investigator.

If the investigator becomes aware of a serious adverse event with a suspected causal relationship to the investigational medicinal product that occurs after the end of the clinical trial in a subject treated by him or her, the investigator shall, without undue delay, report the serious adverse event to the sponsor.

13.3 Follow-up of adverse events

All AEs will be followed until they have abated, or until a stable situation has been reached. Depending on the event, follow up may require additional tests or medical procedures as indicated, and/or referral to the general physician or a medical specialist. SAEs need to be reported until the end of trial (as defined in Chapter 12.1).

13.4 Reporting of SUSARs by the sponsor to EudraVigilance

The sponsor will keep detailed records of all AEs which are reported to him/her by the investigator or investigators. The sponsor will report electronically and without delay to EudraVigilance all relevant information about any SUSAR.

The period for the reporting of SUSARs by the sponsor to EudraVigilance will take account of the seriousness of the reaction and will be as follows:

- In the case of <u>fatal or life-threatening</u> SUSARs, as soon as possible and in any event not later than **7 days** after the sponsor became aware of the reaction;
- In the case of <u>non-fatal or non-life-threatening</u> SUSARs, not later than **15 days** after the sponsor became aware of the reaction;
- In the case of a SUSARs which was initially considered to be non-fatal or nonlife threatening but which turns out to be fatal or life-threatening, as soon as possible and in any event not later than **7 days** after the sponsor became aware of the reaction being fatal or life-threatening.

Where necessary to ensure timely reporting, the sponsor may, in accordance with section 2.4 of Annex III, submit an initial incomplete report followed up by a complete report.

13.5 Annual safety report

Regarding investigational medicinal products other than placebo, the sponsor shall submit annually through CTIS to all Member States concerned a report on the safety of each investigational medicinal product used in a clinical trial.

13.6 Unblinding procedures for safety reporting

The investigator will only unblind the treatment allocation of a subject in the course of a clinical trial if unblinding is relevant to the safety of the subject.

When reporting a SUSAR to the EMA, the sponsor will only unblind the treatment allocation of the affected subject to whom the SUSAR relates.

In case of unblinding, describe procedure to maintain blind for persons responsible for the ongoing conduct of the clinical trial such as the management, monitors, investigators) and those persons responsible for data analysis and interpretation of results at the conclusion of the clinical trial, such as biometrics personnel.

Unblinded information will be accessible only to persons who need to be involved in the safety reporting to the EMA or to persons performing ongoing safety evaluations during the clinical trial.

13.7 Temporary halt for reasons of subject safety

The sponsor will suspend the study if there is sufficient ground that continuation of the study will jeopardise subject health or safety. The sponsor will submit the notification through CTIS without undue delay of a temporary halt but not later than in 15 days of the date of the temporary halt. It shall include the reasons for such action and specify follow-up measures. The study will be suspended pending a further positive decision by the concerned member state. The investigator will take care that all subjects are kept informed.

13.8 Urgent safety measures and other relevant safety reporting

Where an unexpected event is likely to seriously affect the benefit-risk balance, the sponsor and the investigator will take appropriate urgent safety measures to protect the subjects. In addition the sponsor will notify the Member States concerned, through CTIS, of the event and the measures taken. That notification will be made without undue delay but no later than **7 days** from the date the measures have been taken.

13.9 Data Safety Monitoring Board (DSMB)/Data Monitoring Committee (DMC)

Not applicable, given that the safety profile of the investigational product is well-documented in literature and is already standard of care (see Chapter 4 and 8).

14. STATISTICAL ANALYSIS

14.1 Description of statistical methods

Statistical analysis will be performed using IBM SPSS Statistics (IBM Corporation and its licensors, 2017. Armonk, NY). The collected data will consist of both categorical and continuous variables. Categorical data will be presented as frequencies and percentages, while continuous variables will be presented as means, standard deviations, medians, and interquartile ranges, as appropriate. If applicable, derived parameters will be calculated based on the primary and secondary outcome measures using appropriate formulas and statistical procedures. All tests will be performed two-sided.

14.2 Randomisation and blinding

Eligible patients who signed informed consent will be randomised by the participating investigators. Randomisation will be performed by a central automatic randomisation tool with an intervention:control ratio of 1:1. Each randomised patient is assigned a sequential subject number.

The investigational product used in this study is a CWI system prepared with bupivacaine or physiological saline based on the allocated treatment arm. Preparation of the investigational product involves the in-hospital relabelling of hospital-acquired medication. The CWI-system will be clearly labelled as containing CIMICS-trial medication with the patient's name and date of birth, and delivered to the preoperative holding. This procedure ensures that patients as well as treating physicians and nursing staff are blinded for the randomisation.

Sufficient reason to unblind to patient allocation is left to the discretion of the treating physician. The logged medication can be found within the digital patient database if the treating physician explicitly indicates a necessity for this. The treating physician will inform the participating investigators of any unblinding.

14.3 Sample size

The primary endpoint of this study is the postoperative QoR score. We hypothesize that the addition of CWI will lead to a clinically significant increase in the QoR score. Based on previous studies, a clinically significant and relevant difference can be demonstrated with a difference in the QoR score of 6 points (with an SD of 14 points).[21,22]

Using this cut-off value for a sample size calculation with a Mann-Whitney U-test to account for possible non-normally distributed data, we need 91 patients per randomization group to achieve 80% power with an alpha of 0.05 in order to detect a clinically relevant difference in QoR score between CWI with local analgesics and placebo. Since we expect a drop-out rate of 5% (e.g. conversion to open surgery), we aim to include 96 patients per group, or 192 patients in total.

14.4 Primary analysis

The primary objective of this study is to compare QoR score between the interventional and control arm as measured on postoperative day 2. Primary outcome analysis will be done using unpaired t-tests or Mann-Whitney U tests, depending on the distribution of the data. A p-value of <0.05 will be considered statistically significant.

14.5 Secondary analysis

Secondary outcome parameters for this study include postoperative QoR score on postoperative day 1 to day 5, postoperative pain levels, postoperative opioid consumption, postoperative length of stay and time until functional recovery, incidence of postoperative complications, markers of recovery, cost-effectiveness, as well as adherence to ERAS protocol and factors associated with increased

opioid use. A p-value of <0.05 will be considered statistically significant. Depending on the distribution of data;

- For continuous variables as measured on postoperative day 2 only, the following statistical tests will be used as appropriate depending on the data distribution: unpaired t-tests for normally-distributed continuous data, while Mann-Whitney U tests for not-normally distributed continuous data;
- For continuous variables as measured at one timepoint only, such as length of stay, the following statistical tests will be used as appropriate depending on the data distribution: unpaired t-tests for normally-distributed continuous data, Mann-Whitney U tests for not-normally-distributed continuous data;
- For continuous variables measured on postoperative day 1 to day 5, or otherwise repeated measures, the following statistical tests will be used as appropriate depending on the data distribution: linear mixed model analyses for normally-distributed data, Friedman tests for not-normally distributed data;
- For categorical variables, the following statistical tests will be used as appropriate depending on the data distribution: chi-squared tests for normally-distributed data, Fisher's exact tests for not-normally distributed data.

Postoperative QoR score on postoperative day 1 to day 5: Postoperative QoR score will be collected daily using the QoR-15 scale. Postoperative QoR scores will be compared between the interventional and control arm as measured on postoperative day 1 to 5. Depending on the distribution of data, a linear mixed model analysis or the Friedman test will be used for comparison. To account for multiplicity, p-value will be adjusted to 0.01, as is in line with the Bonferroni method.

Postoperative pain levels on postoperative day 2: Pain levels will be assessed using the Numeric Rating Scale (NRS), and will be compared between the interventional and control arm as measured on postoperative day 2 only. Depending on the distribution of data, unpaired t-tests or Mann-Whitney U tests will be used for comparison. A p-value of <0.05 will be considered statistically significant.

Postoperative pain levels on postoperative day 1 to day 5: Pain levels will be assessed using the Numeric Rating Scale (NRS), and will be compared between the interventional and control arm as measured on postoperative day 1 to 5. Depending on the distribution of data, a linear mixed model analysis or the Friedman test will be used for comparison. To account for multiplicity, p-value will be adjusted to 0.01, as is in line with the Bonferroni method.

Postoperative opioid use: Total opioid consumption will be recorded in milligrams (mg) of morphine equivalents, and compared between the interventional and control arm. Depending on the distribution of data, unpaired t-tests or Mann-Whitney U tests will be used for comparison. A p-value of <0.05 will be considered statistically significant.

Postoperative length of stay and Time until functional recovery: Postoperative length of stay and time until functional recovery will be measured in days, and compared between the interventional and control arm. Based on the data distribution, unpaired t-tests will be applied for normally distributed continuous variables, and Mann-Whitney U tests will be used for non-normally distributed continuous variables. A p-value of <0.05 will be considered statistically significant.

Incidence of CWI catheter-related and overall postoperative complications: CWI catheter-related and overall complications within 30 days postoperatively will be noted and classified according to Clavien-Dindo. These outcomes will be recorded as categorical data and compared between the interventional and control arm using Chi-squared or Fisher's exact tests, based on the data distribution. A p-value of <0.05 will be considered statistically significant.

Markers of recovery:

a. Time to first flatulence, time to stool passage, and time to mobilization will be measured in days, and compared between the interventional and control arm. Based on the data distribution, unpaired t-tests will be applied for normally distributed continuous variables, and Mann-Whitney U tests will be used for non-normally distributed continuous variables. A p-value of <0.05 will be considered statistically significant.

d. Condition at discharge will be noted and classified according to the WHO-classification, and will be compared between the interventional and control arm using Chi-squared or Fisher's exact tests, as appropriate. A p-value of <0.05 will be considered statistically significant.

Adherence to the ERAS protocol: Compliance to the ERAS protocol will be measured by scoring adherence to each ERAS element with a yes or no. The percentage of ERAS elements followed in the patient's peri-operative care is then calculated to determine overall compliance, as well as pre-, intra-, and post-operative compliance. This will be compared between the interventional and control arm using descriptive statistics and the appropriate statistical tests based on the data distribution; unpaired t-tests will be applied for normally-distributed continuous variables, while Mann-Whitney U tests will be used for non-normally distributed continuous variables. A p-value of <0.05 will be considered statistically significant.

To assess the impact of the duration of CWI infusion on recovery outcomes on postoperative day 2: Duration of CWI infusion will be measured in days. Postoperative QoR score will be scored and recorded using the daily QoR-15 questionnaire. An ANCOVA (Analysis of Covariance) will be performed with the length of CWI infusion as a covariate to adjust for differences in infusion duration among patients. This approach will help isolate the effect of the interventional treatment on postoperative day 2 QoR score by accounting for the variation in infusion times.

14.6 Procedure for accounting for missing, unused and spurious data

All the collected data is expected to be used. Missing data will be handled using the Intention to Treat (ITT) approach. All participants will be included in the analysis as per their original group assignments, regardless of adherence to study protocol or completion status. This method ensures that the randomization benefits are preserved, and will provide a realistic estimate of the treatment effect.

15. ETHICAL CONSIDERATIONS

15.1 Declaration of Helsinki

The study is conducted according to the principles of the Declaration of Helsinki (64th WMA General Assembly, Fortaleza, Brazil, October 2013) and in accordance with the Medical Research Involving Human Subjects Act (WMO), the Good Clinical Practice (ICH-GCP) guidelines and the General Data Protection Regulation (in Dutch AVG).

15.2 Recruitment and informed consent procedures

Informed consent will be obtained prior to any study related procedures being undertaken at screening (see Chapter 11). Informed consent will be written, dated and signed by the person performing the interview, and by the subject. The investigator or his/her representative will explain the nature of the study to the subject, and answer all questions regarding this study. In the interview it will be verified that the subject has understood the information. The subject will be provided with a copy of the document (or the record) by which informed consent has been given. The informed consent will be documented. Adequate time will be given for the to consider his or her decision to participate in the clinical trial.

15.3 Benefits and risks assessment, group relatedness

All interventional products will be used in the authorised form for the authorised indication. Their safety profiles are well-documented and well-established. Possible side effects of the CWI system include mild pain, redness or swelling at the infusion site as well as a very low risk of infection at the site of the infusion.[23] According to earlier studies, the risk of wound infections is not increased in patients who receive CWI.[24] It should be noted that all interventional products are already used as part of standard care. Participating in this study does not expose patients to an additional risk.

As a low-intervention clinical trial, the focus of this research will be analysing the effectiveness of both interventions as an addition to current clinical practice, as both interventions are widely used without scientific substantiation for superiority or inferiority of either treatment, compared to placebo. The additional risk to participating patients is therefore considered minimal.

In terms of benefits, additional treatment in the interventional groups may reduce patient postoperative opioid consumption and accelerate recovery, which would be beneficial for the patients' comfort and overall outcomes.

In conclusion, the investigators consider the extra potential risk for patients in the interventional arms to be minimal and expect it to be outweighed by the potential benefits

15.4 Compensation for injury

The sponsor has an insurance that is in accordance with the legal requirements in the Netherlands (Article 7 WMO, under 1). This insurance provides cover for damage to research subjects through injury or death caused by the study. The insurance applies to the damage that becomes apparent during the study or within 4 years after the end of the study.

The sponsor/investigator has a liability insurance that is in accordance with article 7, under 9, of the WMO.

15.5 Compensation for subjects

Not applicable.

15.6 Compensation for investigators

Not applicable.

16. ADMINISTRATIVE ASPECTS, MONITORING AND CONFIDENTIALITY

The study will be conducted in compliance with the protocol, with Clinical Trials Regulation No 536/2014 and with the principles of good clinical practice.

16.1 Approval initial application and substantial modifications

The trial protocol, informed consent form, subject information leaflet, investigational medicinal product dossier, investigators brochure and any other documents required by the Regulation will be submitted for the regulatory approval before the clinical trial is started via CTIS.

The sponsor will also submit and obtain approval for substantial modifications to the original approved documents via CTIS.

A ‘substantial modification’ is defined in the CTR as any change to any aspect of the clinical trial which is made after notification of a decision referred to in Articles 8, 14, 19, 20 or 23 and which is likely to have a substantial impact on the safety or rights of the subjects or on the reliability and robustness of the data generated in the clinical trial.

16.2 Monitoring

Given that this is a low-intervention clinical trial, monitoring will be conducted by an independent monitor from the participating hospital using a risk-based approach.

16.3 Recording, handling and storage of information

16.3.1 Handling of data and data protection

Individual patient information obtained as a result of this study is considered confidential and is handled conform the Dutch Personal Data Protection Act (AVG) and the General Data Protection Regulation (EU) 2016/679. Patients’ confidentiality will be ensured by using study numbers. Every randomised patient is assigned a study number. Communication occurs only with this number. A subject identification code list is safeguarded by the coordinating investigator. Disclosure to third parties is prohibited, except for authorities involved with monitoring and quality control of the study.

All data required to answer the primary and secondary objectives will be collected using only study numbers in an ISO 27001 certified study database with an electronic case report form (eCRF). Data management is performed by the local investigator. All source documents should be completed in a neat, legible manner to ensure accurate interpretation of data.

16.3.2 Source documents and case report forms (CRF)

Source documents for this study will include hospital records and procedure reports and data collection forms. These documents will be used to enter data on the CRFs. Data reported on the CRF that are derived from source documents must be consistent with the source documents or the discrepancies must be explained.

All documents will be stored safely in confidential conditions. On all study-specific documents other than the signed consent, the subject will be referred to by the study subject identification code.

Information regarding postoperative recovery will be collected from the ISO 27001 certified ERAS Interactive Audit System (EIAS). Patients additionally complete daily questionnaires digitally by using an ISO 27001 certified information security system. Local researchers will collect the digital results from the patient file and the database.

16.3.3 Clinical trial master file and data archiving

The sponsor and the investigator shall keep a clinical trial master file. The clinical trial master file shall at all times contain the essential documents relating to the clinical trial which allow verification of the conduct of a clinical trial and the quality of the data generated.

The sponsor and the investigator shall archive the content of the clinical trial master file for at least 25 years after the end of the clinical trial, unless other EU law requires archiving for a longer period. The medical files of subjects shall be archived in accordance with national law.

Archiving of documents will be continued until the project has finished. These documents will be stored for a maximum duration of 25 years after completion of the study. These data will be accessible to the current and future members of the research team. Patients who do not agree with these terms, are excluded from participation.

16.4 Audits and inspections and direct access to source data/documents

This trial may be subject to internal or external monitoring, auditing or inspections procedure to ensure adherence to GCP. Access to all trial-related documents including direct access to source data will be given at that time.

16.5 Reporting of serious breaches

The sponsor will notify the Member States concerned about a serious breach of the Regulation or of the version of the protocol applicable at the time of the breach through CTIS without undue delay but not later than **seven days** of becoming aware of that breach.

16.6 Data breach response procedures

In the event of a data security breach, the sponsor will implement a series of measures to prevent or reduce further impact. These measures will at minimum include:

- The sponsor will prioritise immediate containment, i.e. will stop the data breach if it is ongoing. Additionally, the sponsor will take efforts to mitigate and limit the damage as applicable, for example through remotely wiping or encrypting compromised devices, or removing published files from online platforms.
- The sponsor will assess the breach to determine the cause and scope. This may include identifying what type of data was involved, the number of affected individuals, and whether the data has been accessed by unauthorized parties.
- The sponsor will promptly notify and report the incident to the Dutch Data Protection Authority within 72 hours of detection. This will be accomplished via the designated national data leak reporting desk. In the event of a serious breach, the sponsor will additionally notify the Member States affected by the breach. This will be done through the Clinical Trials Information System (CTIS) in accordance with section 16.5 of the study protocol.
- Should the data breach present a high risk to the individuals participating in the study, the affected subjects will also be informed without delay. This notification will include detailed information about the nature of the breach, potential consequences, and recommended protective measures, ensuring that study subjects are fully aware and can take appropriate actions to safeguard their personal information.
- The sponsor will record all breaches in a data breach register, documenting the cause, affected data, consequences, and remedial actions taken, to learn from past incidents and improve future data security measures.

16.7 Notification of the start and the end of the recruitment

The sponsor will notify within 15 days each Member State concerned of the start of a clinical trial in relation to that Member State through CTIS.

The sponsor will notify within 15 days each Member State concerned of the first visit of the first subject in relation to that Member State through CTIS.

The sponsor will notify within 15 days each Member State concerned of the end of the recruitment of subjects for a clinical trial in that Member State through the EU.

16.8 Temporary halt/(early) termination

The sponsor will notify within 15 days each Member State concerned of the end of a clinical trial in relation to that Member State through CTIS.

The sponsor will notify within 15 days each Member State concerned of the end of a clinical trial in all Member States concerned and in all third countries in which the clinical trial has been conducted through CTIS.

16.8.1 Temporary halt/early termination for reasons not affecting the benefit-risk balance

The sponsor will notify with 15 days each Member State concerned of a temporary halt of a clinical trial in all Member States concerned for reasons not affecting the benefit-risk balance through CTIS.

When a temporarily halted clinical trial for reasons not affecting the benefit-risk balance is resumed the sponsor will notify each Member State concerned through CTIS.

The sponsor will notify to the EU portal CTIS of early termination of the clinical trial for reasons not affecting the benefit-risk balance through CTIS. The reasons for such action and, when appropriate, follow-up measures for the subjects will be provided as well.

16.8.2 Temporary halt/early termination for reasons of subject safety

In accordance to article 38 of the CTR, the sponsor will suspend the study if there is sufficient ground that continuation of the study will jeopardise subject health or safety. The temporary halt or early termination of a clinical trial for reasons of a change of the benefit-risk balance will be notified to the Member States concerned through the EU portal CTIS without undue delay but not later than in 15 days of the date of the temporary halt or early termination. It shall include the reasons for such action and specify follow-up measures. The restart of the clinical trial following a temporary halt as referred to in paragraph 1 shall be deemed to be a substantial modification subject to the authorisation procedure laid down in Chapter III of the CTR.

16.9 Summary of the results

Within one year from the end of a clinical trial in all Member States concerned, a summary of the results of the clinical trial will be submitted to the EU database CTIS. The content of the summary of the results is set out in CTR Annex IV. It shall be accompanied by a summary written in a manner that is understandable to laypersons. The content of the summary is set out in CTR Annex V.

16.10Public disclosure and publication policy

Within one year after the primary endpoint has been reached, final results of the study will be submitted to an international peer-reviewed journal. The coordinating and participating investigators will prepare the manuscript together with those who substantially contributed to the study. Any publication, abstract or presentation based on patients included in this study must be approved by the coordinating and participating investigators.

17. REFERENCES

1. Gustafsson, U.O., Scott, M.J., Hubner, M., Nygren, J., Demartines, N., Francis, N., Rockall, T.A., Young-Fadok, T.M., Hill, A.G., Soop, M., De Boer, H.D., Urman, R.D., Chang, G.J., Fichera, A., Kessler, H., Grass, F., Whang, E.E., Fawcett, W.J., Carli, F., Lobo, D.N., Rollins, K.E., Balfour, A., Baldini, G., Riedel, B., Ljungqvist, O., 2019. Guidelines for Perioperative Care in Elective Colorectal Surgery: Enhanced Recovery After Surgery (ERAS®) Society Recommendations: 2018. World Journal of Surgery 43, 659–695.. doi:10.1007/s00268-018-4844-y
2. Pisarska, Magdalena et al. "Do we really need the full compliance with ERAS protocol in laparoscopic colorectal surgery? A prospective cohort study." International journal of surgery (London, England) vol. 36,Pt A (2016): 377-382. doi:10.1016/j.ijsu.2016.11.088
3. Li, L., Jin, J., Min, S., Liu, D., Liu, L., 2017. Compliance with the enhanced recovery after surgery protocol and prognosis after colorectal cancer surgery: A prospective cohort study. Oncotarget 8, 53531–53541. doi:10.18632/oncotarget.18602
4. Ripollés-Melchor, Javier et al. "Association Between Use of Enhanced Recovery After Surgery Protocol and Postoperative Complications in Colorectal Surgery: The Postoperative Outcomes Within Enhanced Recovery After Surgery Protocol (POWER) Study." JAMA surgery vol. 154,8 (2019): 725-736. doi:10.1001/jamasurg.2019.0995
5. Simpson, J., Bao, X., Agarwala, A., 2019. Pain Management in Enhanced Recovery after Surgery (ERAS) Protocols. Clinics in Colon and Rectal Surgery 32, 121–128.. doi:10.1055/s-0038-1676477
6. Beverly, Anair et al. "Essential Elements of Multimodal Analgesia in Enhanced Recovery After Surgery (ERAS) Guidelines." Anesthesiology clinics vol. 35,2 (2017): e115-e143. doi:10.1016/j.anclin.2017.01.018
7. Wick EC, Grant MC, Wu CL. Postoperative Multimodal Analgesia Pain Management With Nonopioid Analgesics and Techniques: A Review. JAMA Surg. 2017;152(7):691–697. doi:10.1001/jamasurg.2017.0898
8. Garimella, V., Cellini, C., 2013. Postoperative Pain Control. Clinics in Colon and Rectal Surgery 26, 191–196.. doi:10.1055/s-0033-1351138
9. Carmichael JC, Keller DS, Baldini G et al (2017) Clinicalpractice guidelines for enhanced recovery after colon and rectalsurgery from the American Society of Colon and Rectal Sur-geons and Society of American Gastrointestinal and EndoscopicSurgeons. Dis Colon Rectum 60:761–784
10. Hubner M, Blanc C, Roulin D et al (2015) Randomized clinical trial on epidural versus patient-controlled analgesia for laparoscopic colorectal surgery within an enhanced recovery pathway.Ann Surg 261:648–653
11. Halabi WJ, Kang CY, Nguyen VQ et al (2014) Epidural analgesia in laparoscopic colorectal surgery: a nationwide analysis of use and outcomes. JAMA Surg 149:130–136
12. Borzellino G, Francis NK, Chapuis O et al (2016) Role of epidural analgesia within an ERAS program after laparoscopic colorectal surgery: a review and meta-analysis of randomized controlled studies. Surg Res Pract 2016:7543684
13. Karthikesalingam, Alan et al. "Continuous wound infusion of local anaesthetic agents following colorectal surgery: systematic review and meta-analysis." World journal of gastroenterology vol. 14,34 (2008): 5301-5. doi:10.3748/wjg.14.5301
14. Mungroop TH, Bond MJ, Lirk P, Busch OR, Hollmann MW, Veelo DP, et al. Preperitoneal or subcutaneous wound catheters as al-ternative for epidural analgesia in abdominal surgery: a systematic review and meta-analysis. Ann Surg. 2019;269:252–60. https://doi.org/10.1097/SLA.00000 00000 00
15. Beaussier, Marc et al. "Continuous preperitoneal infusion of ropivacaine provides effective analgesia and accelerates recovery after colorectal surgery: a randomized, double-blind, placebo-controlled study." Anesthesiology vol. 107,3 (2007): 461-8. doi:10.1097/01.anes.0000278903.91986.19
16. Mungroop TH, Bond MJ, Lirk P, Busch OR, Hollmann MW, Veelo DP, et al. Preperitoneal or subcutaneous wound catheters as al-ternative for epidural analgesia in abdominal surgery: a systematic review and meta-analysis. Ann Surg. 2019;269:252–60. https://doi.org/10.1097/SLA.00000 00000 00
17. Fustran, N., Dalmau, A., Ferreres, E., Camprubí, I., Sanzol, R., Redondo, S., Kreisler, E., Biondo, S., Sabaté, A., 2015. Postoperative analgesia with continuous wound infusion of local anaesthesiavssaline: a double-blind randomized, controlled trial in colorectal surgery. Colorectal Disease 17, 342–350.. doi:10.1111/codi.12893
18. Ketelaers, S.H.J., Dhondt, L., Van Ham, N., Harms, A.S., Harm, Nieuwenhuijzen, G.A.P., Rutten, H.J.T., Jacobus, Bloemen, J.G., Vogelaar, F.J., 2022. A prospective cohort study to evaluate continuous wound infusion with local analgesics within an enhanced recovery protocol after colorectal cancer surgery. Colorectal Disease 24, 1172–1183.. doi:10.1111/codi.16201
19. Myles, Paul S et al. "Minimal Clinically Important Difference for Three Quality of Recovery Scales." Anesthesiology vol. 125,1 (2016): 39-45. doi:10.1097/ALN.0000000000001158
20. de Vlieger, J.C.N., Luiting, W.H., Lockyer, J. et al. Validation of the Dutch translation of the quality of recovery-15 scale. BMC Anesthesiol 22, 243 (2022). https://doi.org/10.1186/s12871-022-01784-5

21. Myles, Paul S et al. "Minimal Clinically Important Difference for Three Quality of Recovery Scales." Anesthesiology vol. 125,1 (2016): 39-45. doi:10.1097/ALN.0000000000001158
22. Paul S. Myles, Daniel B. Myles; An Updated Minimal Clinically Important Difference for the QoR-15 Scale. Anesthesiology 2021; 135:934–935 doi: https://doi.org/10.1097/ALN.0000000000003977
23. College ter Beoordeling van Geneesmiddelen (CBG)(n.d.). "Bupivacaïne Eugia 2,5 mg/ml oplossing voor injectie." https://www.geneesmiddeleninformatiebank.nl/nl/rvg20949, consulted on 21-02-2024 at https://www.geneesmiddeleninformatiebank.nl/smpc/h20949_smpc.pdf
24. Ventham NT, Hughes M, O'Neill S, Johns N, Brady RR, Wigmore SJ. Systematic review and meta-analysis of continuous local anaesthetic wound infiltration versus epidural analgesia for postoperative pain following abdominal surgery. Br J Surg. 2013;100:1280–9. https://doi.org/10.1002/bjs.9204
25. European Parliament and the Council of the European Union. (2014). Regulation (EU) No 536/2014 of the European Parliament and of the Council of 16 April 2014 on clinical trials on medicinal products for human use, and repealing Directive 2001/20/EC (32014R0536). Official Journal of the European Union. https://eur-lex.europa.eu/legal-content/EN/TXT/?uri=CELEX%3A32014R0536
26. Shida, D., Wakamatsu, K., Tanaka, Y. et al. The postoperative patient-reported quality of recovery in colorectal cancer patients under enhanced recovery after surgery using QoR-40. BMC Cancer 15, 799 (2015). https://doi.org/10.1186/s12885-015-1799-3

Addendum A

Nederlandstalige QoR-15 vragenlijst

Wilt u het cijfer omcirkelen dat het meest op u van toepassing is?

Voorbeeld:

Ik kon makkelijk uit bed stappen	Geen enkel moment	0	1	2	3	4	(5)	6	7	8	9	10	De hele tijd

(Mag u het gevraagde niet doen, kies dan 0)

DEEL A

Hoe voelde u zich de afgelopen 24 uur?

(0 tot 10, waarbij 0 = geen enkel moment [slecht] en 10 = de hele tijd [uitstekend])

1. Ik kon makkelijk ademhalen	Geen enkel moment	0	1	2	3	4	5	6	7	8	9	10	De hele tijd
2. Ik kon van eten genieten	Geen enkel moment	0	1	2	3	4	5	6	7	8	9	10	De hele tijd
3. Ik voelde me uitgerust	Geen enkel moment	0	1	2	3	4	5	6	7	8	9	10	De hele tijd
4. Ik heb goed geslapen	Geen enkel moment	0	1	2	3	4	5	6	7	8	9	10	De hele tijd
5. Ik kon mezelf zonder hulp wassen en verzorgen	Geen enkel moment	0	1	2	3	4	5	6	7	8	9	10	De hele tijd
6. Ik kon communiceren met familie of vrienden	Geen enkel moment	0	1	2	3	4	5	6	7	8	9	10	De hele tijd
7. Ik voelde me gesteund door de artsen en verpleegkundigen in het ziekenhuis	Geen enkel moment	0	1	2	3	4	5	6	7	8	9	10	De hele tijd
8. Ik kon weer aan het werk of gebruikelijke huishoudelijke activiteiten verrichten	Geen enkel moment	0	1	2	3	4	5	6	7	8	9	10	De hele tijd
9. Ik voelde me prettig en had het gevoel dat ik zelf kon bepalen wat er gebeurde	Geen enkel moment	0	1	2	3	4	5	6	7	8	9	10	De hele tijd
10. Ik voelde me over het algemeen goed	Geen enkel moment	0	1	2	3	4	5	6	7	8	9	10	De hele tijd

DEEL B

In hoeverre heeft u de afgelopen 24 uur last gehad van onderstaande klachten?

(10 tot 0, waarbij 10 = geen enkel moment [**uitstekend**] en 0 = de hele tijd [slecht])

11. Matige pijn	Geen enkel moment	10	9	8	7	6	5	4	3	2	1	0	De hele tijd
12. Hevige pijn	Geen enkel moment	10	9	8	7	6	5	4	3	2	1	0	De hele tijd
13. Misselijkheid of braken	Geen enkel moment	10	9	8	7	6	5	4	3	2	1	0	De hele tijd
14. Bezorgdheid of angst	Geen enkel moment	10	9	8	7	6	5	4	3	2	1	0	De hele tijd
15. Verdrietig of somber gevoel	Geen enkel moment	10	9	8	7	6	5	4	3	2	1	0	De hele tijd

Controleer alstublieft of u **alle** 15 vragen heeft beantwoord. Bedankt voor uw medewerking!

Copyright 2021 Universitair Medisch Centrum Groningen, afdeling Anesthesiologie.

ADDITIONELE VRAGEN

DEEL C
Omcirkel wat voor u van toepassing is.

1. Heeft u in de afgelopen 24 uur overgegeven?

   Nee   Ja, één keer   Ja, meerdere keren

2. Bent u de afgelopen 24 uur misselijk geweest?

   Nee   Ja, soms   Ja, meestal   Ja, altijd

3. Hoeveel uren heeft u de afgelopen 24 uur overdag rondgelopen of gestaan?

   Minder dan 2 uur   2 – 6 uur   6 – 12 uur   Meer dan 12 uur

4. Hoeveel pijn ervaart u? Op deze schaal kunt u een cijfer geven op een schaal van 0 tot 10. U geeft 0 aan als u helemaal geen pijn heeft en 10 als u de ergste pijn heeft die u zich voor kunt stellen.

   0   1   2   3   4   5   6   7   8   9   10

5. Heeft u de afgelopen 24 uur pijnstilling medicatie ingenomen?

   Nee   Ja, paracetamol   Ja, paracetamol en extra pijnmedicatie, namelijk…

6. Maakt u nog gebruik van de pomp met lokale pijnstilling?

   Ja, op stand 8   Ja, op stand 5   Ja, op stand 3   Nee, die is verwijderd

7. Is uw stoelgang op gang?

   Ik heb ontlasting gehad   Ik laat windjes   Mijn buik rommelt   Nee, ik voel nog niets
